# Brunneroma: A Rare Cause of Duodeno-duodenal Intussusception

**DOI:** 10.5005/jp-journals-10018-1174

**Published:** 2016-07-09

**Authors:** Aparna M Patankar, Anju M Wadhwa, Aneeta Bajaj, Amol Ingule, Prasad Wagle

**Affiliations:** 1 Department of Radiology , Lilavati Hospital and Research Centre, Mumbai, Maharashtra , India; 2 Department of Gastrointestinal Surgery, Lilavati Hospital and Research Centre, Mumbai, Maharashtra, India

**Keywords:** Brunner gland hamartoma, Brunneroma, Duodenal intussusception, Duodenal polyp, Endoscopic sonography, Intussuscepting duodenal mass on computed tomography, Submucosal duodenal lesion.

## Abstract

Brunner gland hamartoma (brunneroma) is a rare benign tumor of the duodenum. It is usually asymptomatic and detected incidentally by endoscopy or other imaging modality. The definitive diagnosis is based on histopathological findings. These may mimic tumors of other natures, such as gastrointestinal stromal tumors (GIST), carcinoids, lipomas, and leiomyomas. Here, we present a case of duodenal polyp presenting with abdominal pain and obstructive symptoms that caused duodenal intussusception. It was surgically removed and found to be a brunneroma on histopathology.

**How to cite this article:**

Patankar AM, Wadhwa AM, Bajaj A, Ingule A, Wagle P. Brunneroma: A Rare Cause of Duodeno-duodenal Intussusception. Euroasian J Hepato-Gastroenterol 2016;6(1):84-88.

## INRODUCTION

Brunner gland hamartoma, also known as brunneroma, is a rare benign tumor of the duodenum (Table 1). These benign tumors can be asymptomatic or can present with obstructive symptoms and/or gastrointestinal bleeding. Intussusception is a rare presentation for a brunneroma with less than 200 cases reported in the literature. Although brunneroma is a histopathological diagnosis, imaging modalities like computed tomography (CT) and endoscopic ultrasound (EUS) help in ruling out other etiologies and aid in surgical planning.

**Table 1:** **Table 1:** Differential diagnosis for duodenal masses

*Sl.no.*	*Condition*	*Most common location*	*Imaging features*	*Other charecteristics*
1	Adenoma	Duodenum	Well circumscibed, homogenously enhancing polypoidal , soft tissue lesion which can be sessile or pedunculated.	Multiple polyps could be associated with Peutz-Jeghers syndrome.
				A large polyp can act as a lead point for intussuception.
2.	Lipoma	Ileum (at IC junction)	fat density, non enhancing.	–
3	Leiomyoma	Jejunum	Homogenously enhancing well-circumscribed lesion.	Can undergone malignant degeneration (leiomyosarcoma) with spread to surrounding lymph nodes.
4.	GIST	Stomach followed by small bowel.	Most commonly appear as exophytic heterogenously enhancing masses with areas of haemorrhage, necrosis, calcification or cystic degeneration. Rarely they may present as homogenously enhancing intraluminal mass. Liver metastasis when present appear hypervascular.	Small bowel GIST are more likely to be malignant.
				Lymph node metastasis is rare.
				Post chemotherapy the liver metastasis become hypovascular.
5	Carcinoid	Distal ileum	Hyper vascular intensely enhancing on arterial phase. Primary tumors are generally small in size. Metastatic lesions also shows intense arterial enhancement. They can appear as mesenteric masses with intense desmoplastic reaction and calcification.	They can be associated with carcinoid syndrome with symptoms like abdominal pain, flushing, diarrhoea. It can cause restrictive cardiomyopathy due to serotonin induced fibrosis.
				^68^GA-DOTA is superior in detecting carcinoids than conventional CT.
6	Lymphoma	Terminal ileum	Homogenously enhancing circumferencial thickening with aneurysmal dilatation of the bowel .Lymph nodal involvement.	Multiple bulky lymph nodal involvement, splenomegaly are differentiating points of lymphoma from adenocarcinoma. Less commonly can appear as polypoidal intraluminal masses causing intussusception.
7	Adeno-carcinoma	Duodenum	Asymmetric ,nodular wall thickening with shouldering of the margin. It shows heterogenous post contrast enhancement.	Local lymph nodal metastasis is common.
				Risk factors associated include- HNPCC
				FAP, celiac disease, crohns disease.

## CASE REPORT

A 31-year-old female of Indian ethnicity came to the gastrointestinal surgery department with complaints of epigastric pain for the last 15 to 20 days. The pain was moderate in intensity, intermittent, and aggravated by eating food (both solids and liquids). It was associated with bouts of nonbilious, non-projectile vomiting. There was no history of hematemesis, melena, jaundice, weight loss, or anorexia. There was no significant medical or surgical history of past illness. On examination, there was mild epigastric tenderness. However, there was no palpable mass, guarding, or rigidity. Her vital signs were stable. The blood parameters were within normal range.

**Figs 1A to D:**
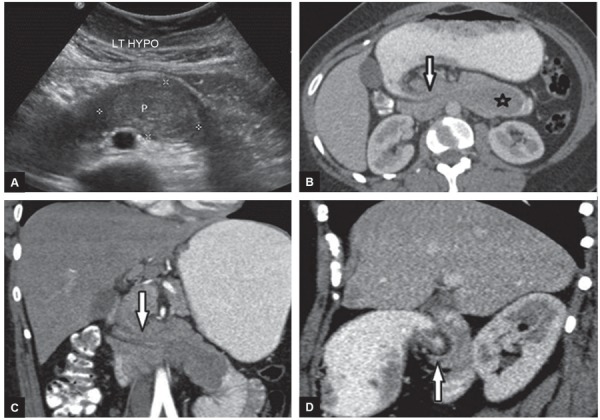
Radiosopic features of Brunner gland hamartoma

An initial ultrasound was performed and a 4.4 × 3 × 2.7 cm oval, well-defined, isoechoic, intraluminal solid mass was found in the region of distal duodenum extending up to the duodeno-jejunal flexure (Fig. 1A). On color Doppler examination, intralesional vascularity was noted.

A multidetector triphasic CT scan of the abdomen and pelvis was performed on Siemens definition flash CT scanner with 5 mm slice thickness and 128 × 0.6 mm collimation. Retrospective 3D reconstruction of volumetric data was performed to obtain coronal and sagittal images. It revealed a 5 × 3.2 cm , well-defined, smooth marginated, and uniformly enhancing soft tissue lesion in the fourth part of the duodenum extending up to the duodeno-jejunal flexure. There was no evidence of any early arterial enhancement, necrosis, or fat density areas within.

The lesion showed a long, slender stalk that appeared to arise from the first part of the duodenum. There was telescoping of the first part of the duodenum into the second part (Figs 1B to D). There was resultant duodenal obstruction with significant dilatation of the stomach; however, the oral contrast passed across the duodenum and was opacifying the small bowel loops. There was no extra luminal component of the mass. There was no significant associated lymphadenopathy. The rest of the abdomen was unremarkable.

**Figs 2A to C:**
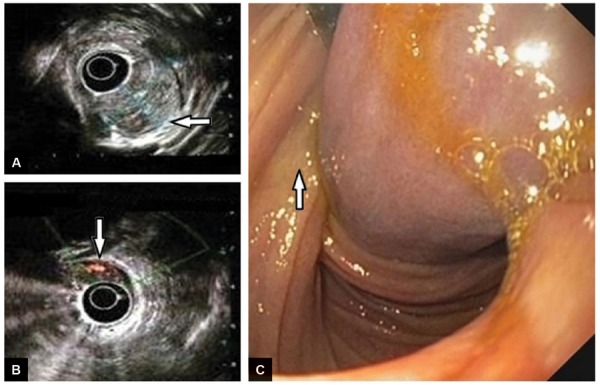
Features of Brunner gland hamartoma

The patient underwent upper gastrointestinal endoscopy with EUS, which revealed a giant polypoidal lesion arising from the junction of the first and second parts of the duodenum with ulcerations at its tip (Figs 2A to C). The EUS revealed approximately 4 × 2.8 × 2 cm large, mixed echoic duodenal submucosal lesion with a thick pedicle, leading to duodenal intussusception and obstruction. Endoscopic ultrasound also identified a feeding artery and possibility of GIST was suggested (Figs 2A to C).

**Figs 3A to D:**
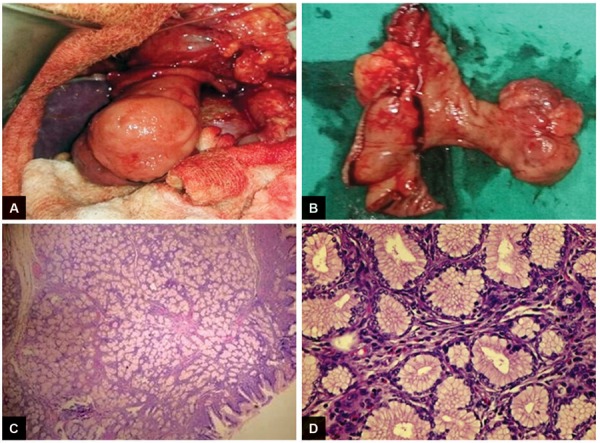
Macroscopic and microscopic views of Brunner gland hamartoma

In view of broad-based pedicle and possibility of GIST, a supra-ampullary duodenectomy was performed .The polypoidal duodenal mass lesion (Figs 3A and B) was removed. The frozen section was suggestive of a brunneroma. The final histopathology report confirmed the frozen section findings. It showed a lobular proliferation of benign submucous (Brunner’s) glands, accompanied by few ducts and scattered stromal elements. The center of stalk was fibrovascular. There was no evidence of dysplasia or malignancy (Figs 3C and D).

## DISCUSSION

Although the small intestine constitutes 75% of the gastrointestinal tract, small intestinal tumors are extremely rare (5%). In the small intestine, duodenal tumors are more common than jejunal and ileal tumors. Adenomas are most common duodenal tumors including adenomatous polyps and Brunner’s adenomas.^1-3^ These glands are present in the first and second parts of the duodenum, and their concentration decreases along with passage from pylorus. In 1835, Curveilheir described the first Brunner gland adenoma.^4^ The American Institute of Radiologic Pathology uses the terms “Brunner gland hyperplasia” for lesions < 5 mm in size and “Brunner’s gland hamartoma” for lesions > 5 mm in size. It has been suggested that Brunner gland hamartoma is better than adenoma because of the following facts: (1) Absence of dysplasia, (2) lack of encapsulation, and (3) admixture of tissues like ducts, acini, smooth muscle cells, adipose tissue, lymphoid tissue, and Brunner gland within a single pathologic lesion. The most common location for brunneroma is the posteromedial wall of the duodenal bulb (70%), the second part of the duodenum (26%), and the third part of the duodenum (4%). They can be pedunculated or sessile.^5,6^

The common age is the 5th to 6th decade of life without any gender predilection. Since Brunner gland secretions protect the duodenal mucosa from acidity of stomach, it is postulated that hyperacidity causes Brunner gland hyperplasia. Thus, *Helicobacter pylori* is a postulated etiological agent.^7^ Smaller tumors can be asymptomatic; larger ones present with gastrointestinal bleed (45%) and small bowel obstruction (50%).^8^ The average size of hamartoma is around 2 cm, but a giant hamartoma up to 11 cm has also been reported. Intussusception is a rare presenting scenario for a brunneroma with less than 200 cases reported in the literature, as was in our case.^6,7^ Brunneromas are benign entities; however, there have been reports of malignant transformation in them.^9,10^

A high index of suspicion is required while diagnosing brunneroma on imaging. The standard imaging modalities employed include barium meal, ultrasound, CT, EUS, and upper gastroendoscopy. The findings on barium and ultrasound are that of nonspecific benign lesions like stromal tumors, carcinoids, or aberrant pancreatic tissue. A CT scan is done to see the extent of the lesion and determine its relationship to adjacent structures.^11^ Hur et al have described the enhancement pattern in brunneroma as it is isodense to the pancreas on plain scan; it does not show any intense enhancement on arterial phase; thus differentiating it from a carcinoid, hypoattenuating to pancreas on portal phase and homogenous enhancement on venous phase.^12^ Upper GI endoscopy is performed to visualize the external morphology and ulcerations if any and to take biopsies. However, pinch biopsies are inconclusive at times as they fail to reach the submucosa. A EUS described by Weisselberg is the current modality of choice. Brunneroma appears hyperechoic with occasional cysts (due to dilated ducts) noted entirely in the submucosa, most importantly not involving the underlying muscularis mucosa.^13^ By demonstrating the benign appearance and the presence/absence of stalk, it can tell whether resection is possible endoscopically or laparoscopically.

Endoscopic polypectomy is performed for smaller lesions. Laparoscopic removal is done in case of large pedunculated lesions. When the second part of the duodenum is involved or when malignancy is suspected, pancreaticoduodenectomy is performed.^14^

Although brunneroma is a rare entity, it should always be considered as a differential diagnosis of duodenal polypoidal lesions. Larger lesions can cause duodeno-duodenal intussusceptions and can present with obstructive symptoms. Imaging tells the extent of the lesion and its relation to adjacent structures and complications, and thus narrows down the differential diagnosis.
